# Supplementary material for: Desiccation-induced fibrous condensation of CAHS protein from an anhydrobiotic tardigrade
Source: Sci Rep. 2021 Nov 4;11:21328. doi: 10.1038/s41598-021-00724-6 (PMC8569203; doi:10.1038/s41598-021-00724-6)
Supplement: Supplementary file 2 — Supplementary Information 2. [file 41598_2021_724_MOESM2_ESM.pdf]

Supplementary Information

Desiccation-induced fibrous condensation of CAHS protein from an anhydrobiotic tardigrade

Maho Yagi-Utsumi, Kazuhiro Aoki, Hiroki Watanabe, Chihong Song, Seiji Nishimura, Tadashi Satoh, Saeko Yanaka, Christian Ganser, Sae Tanaka, Vincent Schnapka, Ean Wai Goh, Yuji Furutani, Kazuyoshi Murata, Takayuki Uchihashi, Kazuharu Arakawa, and Koichi Kato*

*Koichi Kato
Email: kkato@excells.orion.ac.jp

This PDF file includes:

Figures S1 to S3
Legends for Movies S1 to S5

Other supplementary materials for this manuscript include the following:

Movies S1 to S5

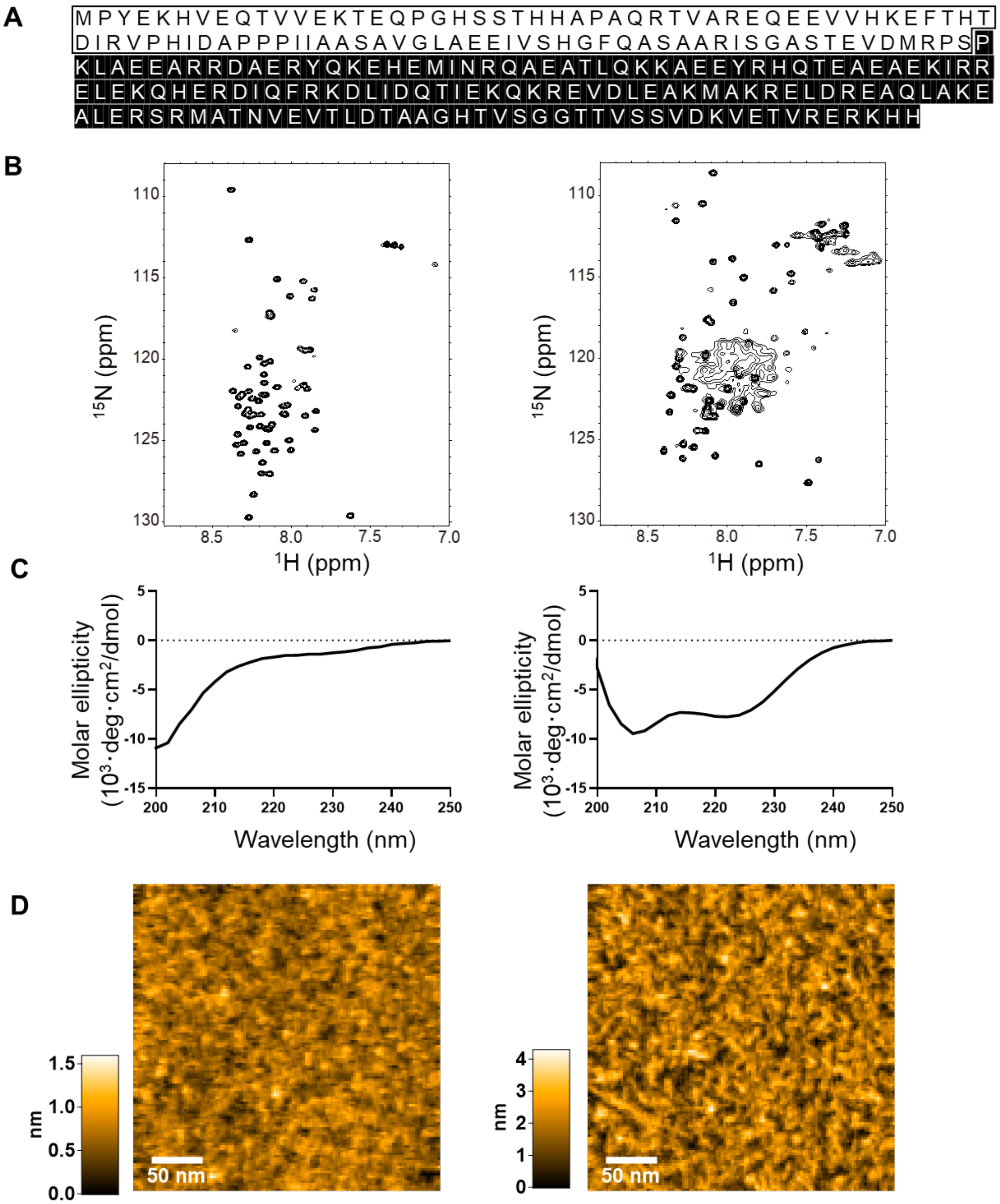

Fig. S1. *In vitro* characterization of CAHS1-N and CAHS1-C. (A) The primary structure of CAHS1 protein with white and black boxes that indicate CAHS1-N and CAHS1-C, respectively. (B) ^{1}H-^{15}N HSQC spectra of CAHS1-N (left) and CAHS1-C (right). (C) CD spectra of CAHS1-N (left) and CAHS1-C (right). (D) Typical HS-AFM images of CAHS1-N (left) and CAHS1-C (right).

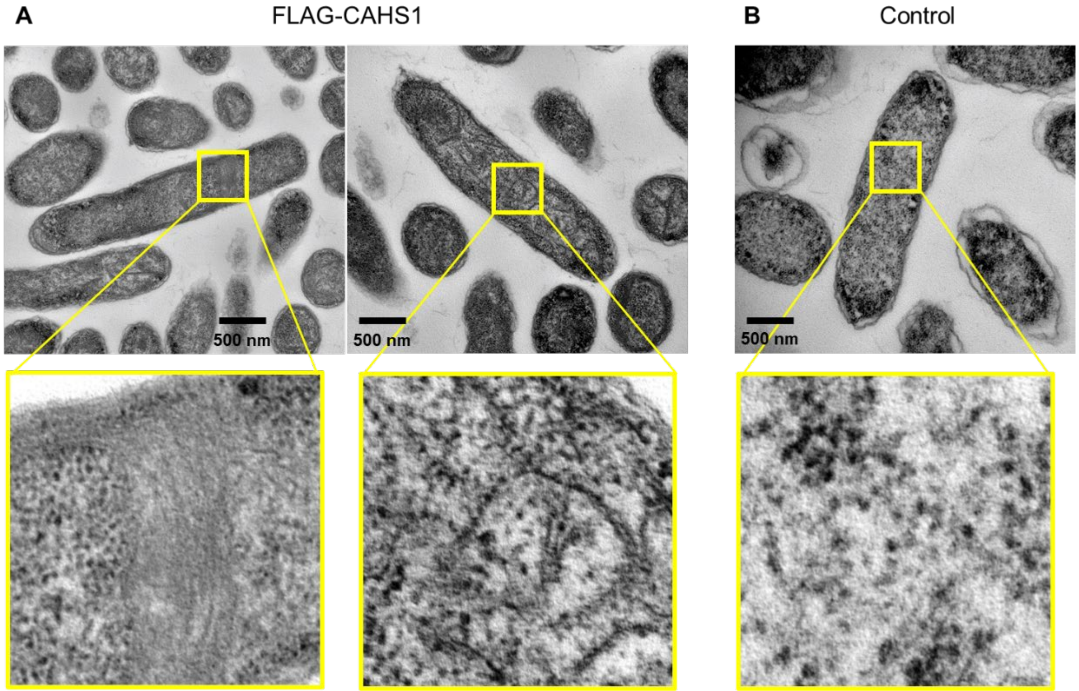

Fig. S2. Fibril formation of the CAHS1 protein in *E. coli*. Cross-sectional TEM images of *E. coli* (A) with and (B) without overexpressed FALG-CAHS1 proteins (top). Enlarged views of the boxed areas of TEM images (bottom).

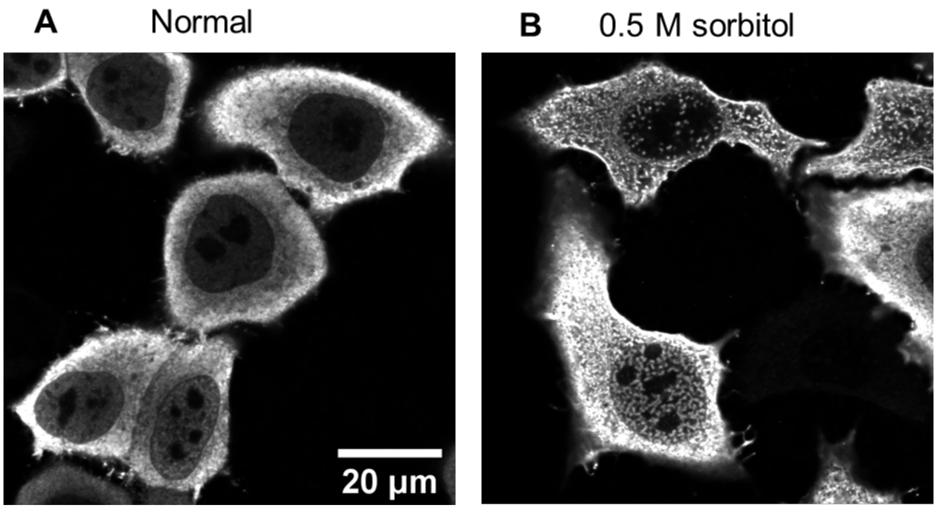

Fig. S3. Osmotic stress induced the CAHS1 protein particles in HeLa cells. The HeLa cells overexpressing the CAHS1-FLAG proteins were exposed to (A) mock or (B) hyperosmotic medium (0.5 M sorbitol) and stained with anti-FLAG antibody.

Movie S1 (separate file). HS-AFM video of a monomeric CAHS1 protein. Scan size, 100 nm × 80 nm. Imaging rate: 0.15 s/frame.

Movie S2 (separate file). HS-AFM video of CAHS1 protein fibril formation. Scan size, 500 nm × 500 nm. Imaging rate: 0.3 s/frame (× 5 play).

Movie S3 (separate file). HS-AFM observation of the disassembly of CAHS1 protein fibrils upon adding 50 mM KCl. Scan size, 200 nm × 160 nm. Imaging rate: 0.2 s/frame (× 2 play).

Movie S4 (separate file). Real-time monitoring of the reversible particle formation of CAHS1-mEGFP protein by a sorbitol-induced osmotic shock. CAHS1-mEGFP (left), mEGFP (middle), and Halo Tag-mEGFP (right).

Movie S5 (separate file). Real-time monitoring of the reversible particle formation of CAHS1-mEGFP protein by a sodium chloride osmotic shock. CAHS1-mEGFP (left), mEGFP (middle), and Halo Tag-mEGFP (right).
